# Tetra­chloridodi-μ_3_-oxido-tetra­kis­(μ_2_-propan-2-olato-κ^2^ *O*:*O*)ditin(II)ditin(IV)

**DOI:** 10.1107/S1600536814000816

**Published:** 2014-01-18

**Authors:** Oleg Yarushnikov, Dina Naumova, Nikolai Klishin, Eduard Rusanov, Oleksii Brusylovets

**Affiliations:** a Department of Chemistry, Kiev National Taras Shevchenko University, Volodymyrska Street 64, 01601 Kiev, Ukraine; b Institute of Organic Chemistry, Murmanskaya Street 4, 253660, Ukraine

## Abstract

The centrosymmetric tetranuclear title molecule, [Sn_4_(C_3_H_7_O)_4_Cl_4_O_2_], contains two types of Sn atoms, Sn^II^ and Sn^IV^. The Sn^II^ atom has a trigonal–pyramidal coordination environment and is bonded to two O atoms from two iso­propano­late groups and one μ_3_-oxide atom. The Sn^IV^ atom has an octa­hedral coordination environment, formed by two chloride atoms, two μ_3_-oxide atoms and two O atoms from iso­propano­late groups.

## Related literature   

For the synthesis and structures of related tin and titanium complexes, see: Boyle *et al.* (2002 ▶); Eslava *et al.* (2010 ▶); Fric & Schubert (2008 ▶); Harrison *et al.* (1978 ▶); Mijatovic *et al.* (2001 ▶); Mokal *et al.* (1994 ▶); Vatsa *et al.* (1991 ▶); Verdenelli *et al.* (2000 ▶).
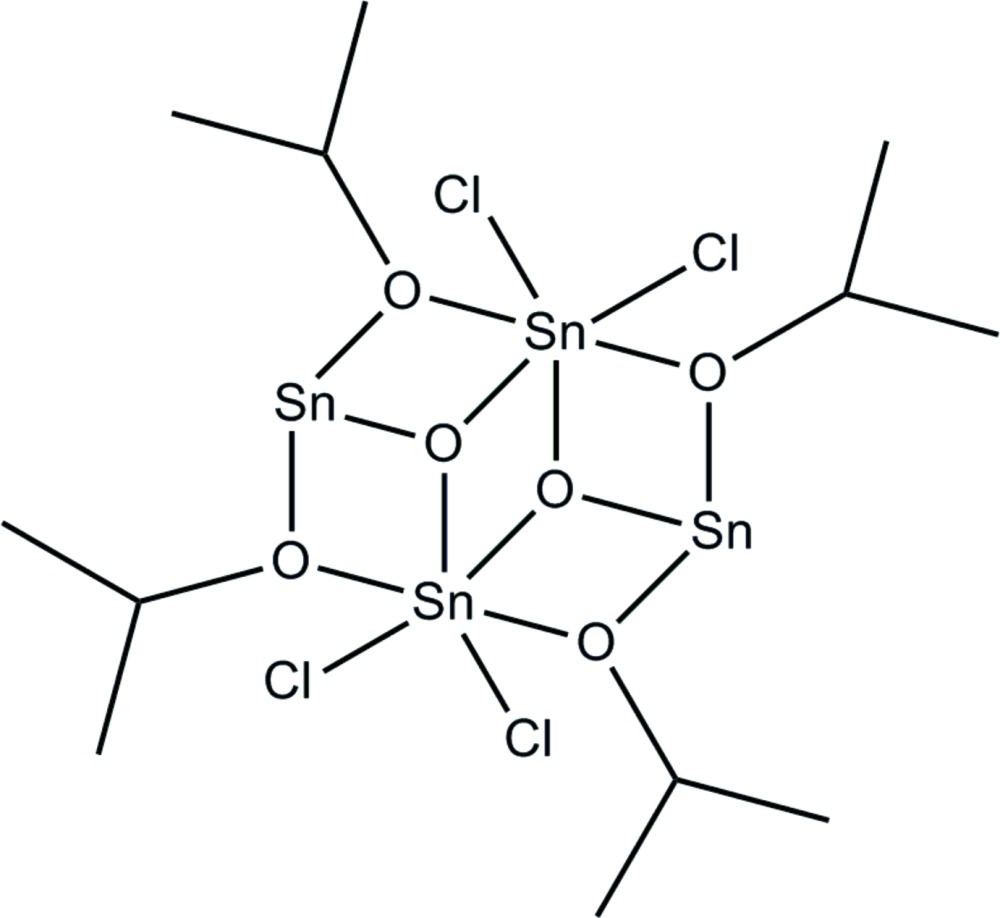


## Experimental   

### 

#### Crystal data  

[Sn_4_(C_3_H_7_O)_4_Cl_4_O_2_]
*M* *_r_* = 884.98
Monoclinic, 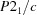

*a* = 6.4423 (3) Å
*b* = 17.8302 (7) Å
*c* = 11.6843 (5) Å
β = 105.474 (2)°
*V* = 1293.5 (1) Å^3^
*Z* = 2
Mo *K*α radiation
μ = 4.25 mm^−1^
*T* = 296 K
0.36 × 0.17 × 0.12 mm

#### Data collection  

Nonius KappaCCD diffractometer
Absorption correction: multi-scan (*DENZO*/*SCALEPACK*; Otwinowski & Minor, 1997 ▶) *T*_min_ = 0.310, *T*_max_ = 0.629
14221 measured reflections
3853 independent reflections
2930 reflections with *I* > 2σ(*I*)
*R*_int_ = 0.041

#### Refinement  

*R*[*F* ^2^ > 2σ(*F* ^2^)] = 0.038
*wR*(*F* ^2^) = 0.061
*S* = 1.10
3853 reflections
118 parameters
H-atom parameters constrained
Δρ_max_ = 0.93 e Å^−3^
Δρ_min_ = −0.80 e Å^−3^

### 

Data collection: *COLLECT* (Nonius, 2000 ▶); cell refinement: *DENZO*/*SCALEPACK* (Otwinowski & Minor, 1997 ▶); data reduction: *DENZO*/*SCALEPACK*; program(s) used to solve structure: *SIR2004* (Burla *et al.*, 2005 ▶); program(s) used to refine structure: *SHELXL97* (Sheldrick, 2008 ▶); molecular graphics: *ORTEP-3 for Windows* (Farrugia, 2012 ▶); software used to prepare material for publication: *SHELXL97*.

## Supplementary Material

Crystal structure: contains datablock(s) I, global. DOI: 10.1107/S1600536814000816/hy2641sup1.cif

Structure factors: contains datablock(s) I. DOI: 10.1107/S1600536814000816/hy2641Isup2.hkl

CCDC reference:

Additional supporting information:  crystallographic information; 3D view; checkCIF report

## Figures and Tables

**Table 1:** Selected bond lengths (Å)

Sn1—O1	2.099 (3)
Sn1—O2	2.084 (3)
Sn1—O3	2.109 (2)
Sn1—O3^i^	2.081 (3)
Sn1—Cl1	2.3808 (12)
Sn1—Cl2	2.3604 (11)
Sn2—O1^i^	2.165 (3)
Sn2—O2	2.168 (3)
Sn2—O3	2.105 (2)

Symmetry code: (i) 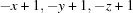.

**Fig. 1.**
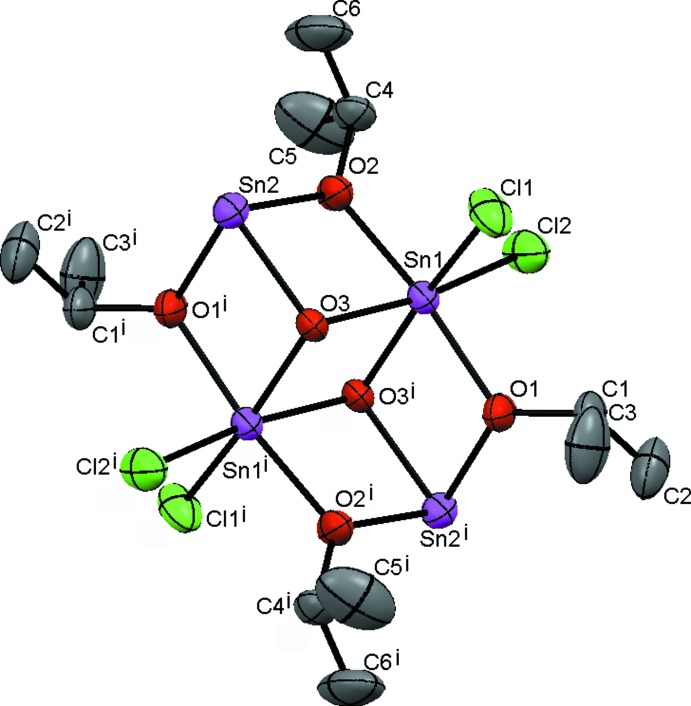
The molecular structure of the title compound, with displacement ellipsoids drawn at the 50% probability level. H atoms have been omitted. [Symmetry code: (i) 1-x, 1-y, 1-z.]

## supplementary crystallographic information

### 1. Comment

Compound with the same symmetry and similar coordination environment as in the title compound was studied in the work of Boyle *et al.* (2002). However it has tin atoms with equal oxidation state, in opposite with our compound, which has different oxidation states and coordination environments of metal atoms. The same situation persists with other compounds of tin and titanium with similar coordination geometry and identical oxidation states of the metal atoms (Eslava *et al.*, 2010; Fric & Schubert, 2008; Harrison *et al.*, 1978; Mijatovic *et al.*, 2001; Mokal *et al.*, 1994; Vatsa *et al.*, 1991; Verdenelli *et al.*, 2000). The title compound has two types of tin atoms. The Sn^II^ atom is three-coordinated and has a trigonal-pyramidal environment. The Sn^IV^ atom is hexa-coordinated and has an octahedral environment. Literature search did not provide any information about similar compounds, which have simultaneously two tin atoms with different oxidation states. In the title compound this is possible due to two chloride atoms for each Sn^IV^ atom.

### 2. Experimental

To a solution of (diisopropoxydichlorido)tin (1.54 g, 5 mmol) in 5 ml of toluene was added N,N,N-tris(trimethylsilyl)aminoiminophosphorane (0.695 g, 2.5 mmol) in 3 ml of toluene and stirred for 4 h. The resulting solution was concentrated to 4 ml and cooled to -25°C. After two days, resulting crystals were filtered from the solution and dried in vacuum (yield: 0.454 g, 41%). Analysis, calculated for C_12_H_28_Cl_4_O_6_Sn_4_: C 16.26, H 3.16, Cl 16.03, O 10.83, Sn 53.72%; found: C 16.01, H 3.56, Cl 16.25, O 10.94, Sn 53.24%.

### 3. Refinement

H atoms were positioned geometrically and refined as riding atoms, with C—H = 0.98 (CH_2_) and 0.96 (CH_3_) Å and with *U*_iso_(H) = 1.2(1.5 for methyl)*U*_eq_(C).

### Crystal data

**Table:**

[Sn_4_(C_3_H_7_O)_4_Cl_4_O_2_]	*F*(000) = 832
*M**_r_* = 884.98	*D*_x_ = 2.272 Mg m^−^^3^
Monoclinic, *P*2_1_/*c*	Mo *K*α radiation, λ = 0.71073 Å
Hall symbol: -P 2ybc	Cell parameters from 3853 reflections
*a* = 6.4423 (3) Å	θ = 2.1–31.1°
*b* = 17.8302 (7) Å	µ = 4.25 mm^−^^1^
*c* = 11.6843 (5) Å	*T* = 296 K
β = 105.474 (2)°	Block, colorless
*V* = 1293.5 (1) Å^3^	0.36 × 0.17 × 0.12 mm
*Z* = 2	

### Data collection

**Table:**

Nonius KappaCCD diffractometer	3853 independent reflections
Radiation source: fine-focus sealed tube	2930 reflections with *I* > 2σ(*I*)
Graphite monochromator	*R*_int_ = 0.041
φ and ω scans with κ offset	θ_max_ = 31.1°, θ_min_ = 2.1°
Absorption correction: multi-scan (*DENZO*/*SCALEPACK*; Otwinowski & Minor, 1997)	*h* = −9→9
*T*_min_ = 0.310, *T*_max_ = 0.629	*k* = −25→21
14221 measured reflections	*l* = −16→15

### Refinement

**Table:**

Refinement on *F*^2^	Primary atom site location: structure-invariant direct methods
Least-squares matrix: full	Secondary atom site location: difference Fourier map
*R*[*F*^2^ > 2σ(*F*^2^)] = 0.038	Hydrogen site location: inferred from neighbouring sites
*wR*(*F*^2^) = 0.061	H-atom parameters constrained
*S* = 1.10	*w* = 1/[σ^2^(*F*_o_^2^) + (0.*P*)^2^ + 2.1387*P*] where *P* = (*F*_o_^2^ + 2*F*_c_^2^)/3
3853 reflections	(Δ/σ)_max_ = 0.001
118 parameters	Δρ_max_ = 0.93 e Å^−^^3^
0 restraints	Δρ_min_ = −0.80 e Å^−^^3^

### Special details

**Table:**

Geometry. All esds (except the esd in the dihedral angle between two l.s. planes) are estimated using the full covariance matrix. The cell esds are taken into account individually in the estimation of esds in distances, angles and torsion angles; correlations between esds in cell parameters are only used when they are defined by crystal symmetry. An approximate (isotropic) treatment of cell esds is used for estimating esds involving l.s. planes.
Refinement. Refinement of F^2^ against ALL reflections. The weighted R-factor wR and goodness of fit S are based on F^2^, conventional R-factors R are based on F, with F set to zero for negative F^2^. The threshold expression of F^2^ > 2sigma(F^2^) is used only for calculating R-factors(gt) etc. and is not relevant to the choice of reflections for refinement. R-factors based on F^2^ are statistically about twice as large as those based on F, and R- factors based on ALL data will be even larger.

### Fractional atomic coordinates and isotropic or equivalent isotropic displacement parameters (Å^2^)

**Table:**

	*x*	*y*	*z*	*U*_iso_*/*U*_eq_	
Sn1	0.47306 (5)	0.589199 (15)	0.51770 (2)	0.02814 (8)	
Sn2	0.07243 (5)	0.494763 (16)	0.33749 (3)	0.03368 (8)	
Cl1	0.1984 (2)	0.65257 (7)	0.58158 (12)	0.0527 (3)	
Cl2	0.6589 (2)	0.69913 (7)	0.49108 (12)	0.0538 (3)	
O1	0.6730 (5)	0.57626 (15)	0.6906 (2)	0.0358 (7)	
O2	0.2858 (5)	0.58942 (15)	0.3422 (2)	0.0347 (7)	
O3	0.2987 (4)	0.48801 (13)	0.5045 (2)	0.0271 (6)	
C1	0.6845 (8)	0.6280 (3)	0.7884 (4)	0.0430 (11)	
H1	0.6029	0.6732	0.7566	0.052*	
C2	0.9156 (9)	0.6503 (3)	0.8431 (5)	0.0674 (17)	
H2A	0.9729	0.6733	0.7838	0.101*	
H2B	0.9987	0.6065	0.8737	0.101*	
H2C	0.9223	0.6852	0.9066	0.101*	
C3	0.5808 (11)	0.5926 (4)	0.8751 (5)	0.083 (2)	
H3A	0.4334	0.5810	0.8360	0.124*	
H3B	0.5860	0.6268	0.9393	0.124*	
H3C	0.6562	0.5474	0.9056	0.124*	
C4	0.2592 (8)	0.6511 (2)	0.2583 (4)	0.0423 (11)	
H4	0.3093	0.6973	0.3024	0.051*	
C5	0.3896 (12)	0.6379 (4)	0.1741 (6)	0.100 (3)	
H5A	0.5384	0.6332	0.2170	0.150*	
H5B	0.3728	0.6794	0.1199	0.150*	
H5C	0.3425	0.5926	0.1304	0.150*	
C6	0.0244 (10)	0.6592 (3)	0.1970 (6)	0.086 (2)	
H6A	−0.0541	0.6678	0.2549	0.130*	
H6B	−0.0269	0.6142	0.1536	0.130*	
H6C	0.0039	0.7009	0.1431	0.130*	

### Atomic displacement parameters (Å^2^)

**Table:**

	*U*^11^	*U*^22^	*U*^33^	*U*^12^	*U*^13^	*U*^23^
Sn1	0.02865 (15)	0.02228 (14)	0.03221 (15)	0.00089 (12)	0.00591 (11)	−0.00050 (12)
Sn2	0.03055 (16)	0.03210 (16)	0.03533 (16)	−0.00024 (13)	0.00347 (12)	0.00033 (13)
Cl1	0.0432 (7)	0.0410 (7)	0.0770 (9)	0.0083 (5)	0.0215 (6)	−0.0119 (6)
Cl2	0.0424 (7)	0.0356 (6)	0.0738 (9)	−0.0129 (5)	−0.0009 (6)	0.0116 (6)
O1	0.0380 (17)	0.0359 (17)	0.0309 (15)	0.0064 (13)	0.0044 (13)	−0.0096 (13)
O2	0.0364 (17)	0.0268 (15)	0.0370 (15)	−0.0058 (13)	0.0032 (13)	0.0050 (13)
O3	0.0297 (15)	0.0200 (14)	0.0302 (14)	0.0016 (11)	0.0058 (11)	−0.0004 (11)
C1	0.053 (3)	0.040 (3)	0.033 (2)	0.007 (2)	0.005 (2)	−0.014 (2)
C2	0.062 (4)	0.076 (4)	0.060 (3)	−0.013 (3)	0.011 (3)	−0.035 (3)
C3	0.103 (5)	0.098 (5)	0.058 (3)	−0.031 (4)	0.042 (4)	−0.035 (3)
C4	0.049 (3)	0.031 (2)	0.042 (3)	−0.003 (2)	0.003 (2)	0.014 (2)
C5	0.137 (7)	0.088 (5)	0.102 (5)	0.023 (5)	0.078 (5)	0.041 (4)
C6	0.079 (5)	0.064 (4)	0.093 (5)	0.004 (3)	−0.017 (4)	0.042 (4)

### Geometric parameters (Å, º)

**Table:**

Sn1—O1	2.099 (3)	C2—H2A	0.9600
Sn1—O2	2.084 (3)	C2—H2B	0.9600
Sn1—O3	2.109 (2)	C2—H2C	0.9600
Sn1—O3^i^	2.081 (3)	C3—H3A	0.9600
Sn1—Cl1	2.3808 (12)	C3—H3B	0.9600
Sn1—Cl2	2.3604 (11)	C3—H3C	0.9600
Sn1—Sn1^i^	3.2382 (5)	C4—C5	1.473 (7)
Sn2—O1^i^	2.165 (3)	C4—C6	1.498 (7)
Sn2—O2	2.168 (3)	C4—H4	0.9800
Sn2—O3	2.105 (2)	C5—H5A	0.9600
O1—C1	1.456 (5)	C5—H5B	0.9600
O2—C4	1.453 (5)	C5—H5C	0.9600
C1—C3	1.494 (7)	C6—H6A	0.9600
C1—C2	1.508 (7)	C6—H6B	0.9600
C1—H1	0.9800	C6—H6C	0.9600
			
O3^i^—Sn1—O2	96.97 (10)	C3—C1—C2	113.2 (4)
O3^i^—Sn1—O1	76.99 (10)	O1—C1—H1	108.3
O2—Sn1—O1	173.28 (10)	C3—C1—H1	108.3
O3^i^—Sn1—O3	78.77 (11)	C2—C1—H1	108.3
O2—Sn1—O3	76.86 (10)	C1—C2—H2A	109.5
O1—Sn1—O3	98.87 (10)	C1—C2—H2B	109.5
O3^i^—Sn1—Cl2	97.63 (8)	H2A—C2—H2B	109.5
O2—Sn1—Cl2	92.84 (8)	C1—C2—H2C	109.5
O1—Sn1—Cl2	90.89 (9)	H2A—C2—H2C	109.5
O3—Sn1—Cl2	168.46 (7)	H2B—C2—H2C	109.5
O3^i^—Sn1—Cl1	164.20 (8)	C1—C3—H3A	109.5
O2—Sn1—Cl1	91.38 (9)	C1—C3—H3B	109.5
O1—Sn1—Cl1	93.83 (8)	H3A—C3—H3B	109.5
O3—Sn1—Cl1	90.17 (8)	C1—C3—H3C	109.5
Cl2—Sn1—Cl1	95.32 (5)	H3A—C3—H3C	109.5
O3^i^—Sn1—Sn1^i^	39.70 (7)	H3B—C3—H3C	109.5
O2—Sn1—Sn1^i^	85.98 (7)	O2—C4—C5	110.1 (4)
O1—Sn1—Sn1^i^	87.47 (7)	O2—C4—C6	108.6 (4)
O3—Sn1—Sn1^i^	39.06 (7)	C5—C4—C6	112.3 (5)
Cl2—Sn1—Sn1^i^	136.34 (4)	O2—C4—H4	108.6
Cl1—Sn1—Sn1^i^	128.33 (3)	C5—C4—H4	108.6
O3—Sn2—O1^i^	75.04 (10)	C6—C4—H4	108.6
O3—Sn2—O2	75.14 (10)	C4—C5—H5A	109.5
O1^i^—Sn2—O2	87.61 (11)	C4—C5—H5B	109.5
C1—O1—Sn1	125.2 (2)	H5A—C5—H5B	109.5
C1—O1—Sn2^i^	127.4 (3)	C4—C5—H5C	109.5
Sn1—O1—Sn2^i^	102.33 (11)	H5A—C5—H5C	109.5
C4—O2—Sn1	126.8 (2)	H5B—C5—H5C	109.5
C4—O2—Sn2	127.8 (2)	C4—C6—H6A	109.5
Sn1—O2—Sn2	102.71 (11)	C4—C6—H6B	109.5
Sn1^i^—O3—Sn2	105.05 (11)	H6A—C6—H6B	109.5
Sn1^i^—O3—Sn1	101.23 (11)	C4—C6—H6C	109.5
Sn2—O3—Sn1	104.02 (10)	H6A—C6—H6C	109.5
O1—C1—C3	109.0 (4)	H6B—C6—H6C	109.5
O1—C1—C2	109.7 (4)		
			
O3^i^—Sn1—O1—C1	162.0 (3)	O1^i^—Sn2—O3—Sn1^i^	−5.91 (11)
O3—Sn1—O1—C1	−121.8 (3)	O2—Sn2—O3—Sn1^i^	−97.42 (13)
Cl2—Sn1—O1—C1	64.4 (3)	O1^i^—Sn2—O3—Sn1	100.06 (12)
Cl1—Sn1—O1—C1	−31.0 (3)	O2—Sn2—O3—Sn1	8.55 (10)
Sn1^i^—Sn1—O1—C1	−159.3 (3)	O3^i^—Sn1—O3—Sn1^i^	0.0
O3^i^—Sn1—O1—Sn2^i^	5.81 (11)	O2—Sn1—O3—Sn1^i^	99.98 (12)
O3—Sn1—O1—Sn2^i^	82.02 (12)	O1—Sn1—O3—Sn1^i^	−74.73 (11)
Cl2—Sn1—O1—Sn2^i^	−91.81 (10)	Cl2—Sn1—O3—Sn1^i^	72.8 (4)
Cl1—Sn1—O1—Sn2^i^	172.79 (10)	Cl1—Sn1—O3—Sn1^i^	−168.64 (9)
Sn1^i^—Sn1—O1—Sn2^i^	44.54 (9)	O3^i^—Sn1—O3—Sn2	−108.82 (14)
O3^i^—Sn1—O2—C4	−112.5 (3)	O2—Sn1—O3—Sn2	−8.83 (11)
O3—Sn1—O2—C4	170.8 (3)	O1—Sn1—O3—Sn2	176.45 (11)
Cl2—Sn1—O2—C4	−14.4 (3)	Cl2—Sn1—O3—Sn2	−36.0 (4)
Cl1—Sn1—O2—C4	81.0 (3)	Cl1—Sn1—O3—Sn2	82.55 (10)
Sn1^i^—Sn1—O2—C4	−150.7 (3)	Sn1^i^—Sn1—O3—Sn2	−108.82 (14)
O3^i^—Sn1—O2—Sn2	85.22 (12)	Sn1—O1—C1—C3	108.1 (4)
O3—Sn1—O2—Sn2	8.53 (10)	Sn2^i^—O1—C1—C3	−101.7 (4)
Cl2—Sn1—O2—Sn2	−176.73 (10)	Sn1—O1—C1—C2	−127.4 (4)
Cl1—Sn1—O2—Sn2	−81.33 (10)	Sn2^i^—O1—C1—C2	22.8 (5)
Sn1^i^—Sn1—O2—Sn2	47.00 (9)	Sn1—O2—C4—C5	102.9 (5)
O3—Sn2—O2—C4	−170.6 (3)	Sn2—O2—C4—C5	−99.2 (5)
O1^i^—Sn2—O2—C4	114.2 (3)	Sn1—O2—C4—C6	−133.8 (4)
O3—Sn2—O2—Sn1	−8.61 (10)	Sn2—O2—C4—C6	24.1 (5)
O1^i^—Sn2—O2—Sn1	−83.76 (12)		

Symmetry code: (i) −*x*+1, −*y*+1, −*z*+1.
